# HIV-Related Discrimination among Grade Six Students in Nine Southern African Countries

**DOI:** 10.1371/journal.pone.0102981

**Published:** 2014-08-08

**Authors:** Brendan Maughan-Brown, Nicholas Spaull

**Affiliations:** 1 Southern Africa Labour and Development Research Unit (SALDRU), University of Cape Town, Cape Town, Western Cape, South Africa; 2 Research on Socioeconomic Policy Group (RESEP), University of Stellenbosch, Stellenbosch, Western Cape, South Africa; London School of Hygiene and Tropical Medicine, United Kingdom

## Abstract

**Background:**

HIV-related stigmatisation and discrimination by young children towards their peers have important consequences at the individual level and for our response to the epidemic, yet research on this area is limited.

**Methods:**

We used nationally representative data to examine discrimination of HIV-positive children by grade six students (n = 39,664) across nine countries in Southern Africa: Botswana, Lesotho, Malawi, Mozambique, Namibia, South Africa, Swaziland, Zambia and Zimbabwe. Descriptive statistics are used to compare discrimination by country, gender, geographic location and socioeconomic status. Multivariate logistic regression is employed to assess potential determinants of discrimination.

**Results:**

The levels and determinants of discrimination varied significantly between the nine countries. While one in ten students in Botswana, Malawi, South Africa and Swaziland would “avoid or shun” an HIV positive friend, the proportions in Lesotho, Mozambique, Zambia and Zimbabwe were twice as high (approximately 20%). A large proportion of students believed that HIV positive children should not be allowed to continue to attend school, particularly in Zambia (33%), Lesotho (37%) and Zimbabwe (42%). The corresponding figures for Malawi and Swaziland were significantly lower at 13% and 12% respectively. Small differences were found by gender. Children from rural areas and poorer schools were much more likely to discriminate than those from urban areas and wealthier schools. Importantly, we identified factors consistently associated with discrimination across the region: students with greater exposure to HIV information, better general HIV knowledge and fewer misconceptions about transmission of HIV via casual contact were less likely to report discrimination.

**Conclusions:**

Our study points toward the need for early interventions (grade six or before) to reduce stigma and discrimination among children, especially in schools situated in rural areas and poorer communities. In particular, interventions should focus on correcting misconceptions that HIV can be transmitted via casual contact.

## Introduction

HIV-related stigmatisation and discrimination by young children towards their peers have important consequences at the individual level and for our response to the epidemic, yet research on this area is limited [1]. Stigma is a risk factor for bullying, victimisation and poorer mental health outcomes among HIV-affected and infected children [2]–[5]. Fear of stigma affects disclosure decisions among children living with HIV [6], and can reduce the desire of children to attend school [7]. Recent qualitative research has also found that fear of stigma may discourage caregivers' disclosure of a child's HIV status to them, resulting in delays in treatment and care for children living with HIV [8]. An understanding of stigmatising attitudes and tendencies to discriminate among young children, as well as the factors that shape these tendencies, is therefore important for the design of stigma reduction interventions. Such interventions can help minimise the negative impact that the epidemic has on the growing numbers of HIV-infected children and their families. Interventions that reduce stigma at an early age may also improve future health outcomes as stigma can influence self-perceived HIV-risk [9], [10], and thus sexual behaviour decisions, and act as a deterrent to HIV-related services, such as HIV-testing [11]–[14].

In this paper, we draw on sociological models of stigma that define stigmatisation as a social process of devaluation within a particular culture or setting, whereby human differences are identified and labelled, and devaluation occurs based on stereotypes associated with those labels [15]–[17]. Discrimination, which is defined as any negative form of distinction, exclusion or restriction affecting an individual [18], is sometimes included as the end point of the stigmatisation process [15]. Here, we situate HIV-related discrimination as a potential outcome of stigmatisation, and since there is not always a direct one-to-one relationship between these concepts [17], we make a clear distinction between them.

Research on attitudes of children towards people living with HIV has focussed on attitudes towards people in general and not specifically towards their peers. Qualitative research in Mali found that 10–13 year old children held many misconceptions about HIV, and a high level of discrimination towards people living with HIV, with a strong link between incorrect knowledge of transmission routes and social stigmatisation [19]. Among fifth grade students in the United States of America, children's desire for a greater social distance from people living with HIV was found to be associated with prejudicial attitudes their mothers held towards homosexuals [20]. In Southern Africa, adolescents in Botswana were found to express a desire for social distance from people living with HIV with the majority of participants having reported that a HIV-positive teacher should not be allowed to teach [21]. In Swaziland, evidence was found among 12–18 year old school students that stigma could be gendered, with boys reporting greater stigmatising attitudes than girls [22]. Their study also concluded that individual and environmental factors shape social stigma, but that individual factors played a more significant role. Cognizant of the need to examine child-on-child stigma specifically [1], a recent study in Zimbabwe explored children's stigmatisation of AIDS-affected children through drawings and stories [23]. While 10–12 year old children in their study were often found to express empathy and respect for AIDS-affected children, findings pointed towards a high degree of stigmatisation with frequent references to bullying, exclusion and desire for social distance.

In this paper we examine discrimination towards HIV-positive children, as measured by the desire for social distance, by grade six students across nine countries in Southern Africa, namely Botswana, Lesotho, Malawi, Mozambique, Namibia, South Africa, Swaziland, Zambia and Zimbabwe. Comparisons between countries contribute to our understanding of variation in discrimination across community contexts and geographical regions [24]. Individual level and contextual factors that may influence discrimination are assessed to increase our knowledge for the targeting of interventions to reduce discrimination and the content of such interventions.

## Methods

### Ethics Statement

The Ministry of Education in each country granted ethical approval for the study and the data is publicly available upon request to The Southern and Eastern African Consortium for Monitoring Educational Quality (SACMEQ) Coordinating Centre (http://www.sacmeq.org/).

### Data

The Southern and Eastern African Consortium for Monitoring Educational Quality (SACMEQ) is a consortium of education ministries, policy-makers and researchers who, in conjunction with UNESCO's International Institute for Educational Planning (IIEP), aims to improve the research capacity and technical skills of educational planners [25], [26] and to provide policy-relevant information on the quality of education in participating countries. To date, it has conducted three nationally representative school surveys in participating countries, specifically SACMEQ I (1996), SACMEQ II (2000), and SACMEQ III (2007). These surveys collected extensive background information on the schooling and home environments of grade 6 students, and tested students and teachers in both numeracy and literacy [27]. In addition, SACMEQ III included a number of attitudinal questions relating to HIV and an HIV-AIDS Knowledge Test (HAKT) comprising 86 true-or-false test items assessing knowledge over five domains: (1) definitions and terminology, (2) transmission mechanisms, (3) avoidance behaviours, (4) diagnosis and treatment, and (5) myths and misconceptions [28]. The SACMEQ student surveys (including the HIV-AIDS Knowledge Test) were written in the same language as the medium of instruction in grade 6 in each country [27]. In most countries this is English, with some exceptions being Mozambique (Portuguese) and Tanzania/Zanzibar (Kiswahili). The surveys were completed by the students themselves.

Our study uses data from SACMEQ III, which interviewed 61,396 grade six students in 2,779 schools in 14 countries, and represents the most recent and comprehensive survey on educational quality in sub-Saharan Africa. The SACMEQ III data is available upon request to the SACMEQ Coordinating Centre (http://www.sacmeq.org/). The SACMEQ sample design was selected so as to meet the standards set down by the International Association of Educational Achievement (IEA) [27]. This ensures that important student level parameters have sampling accuracy at least equivalent to a simple random sample of 400 students, guaranteeing a 95 percent confidence interval of sample means of plus or minus one tenth of a student standard deviation [27]. The SACMEQ III survey used complex two-stage cluster sampling including weighting adjustments to compensate for variations in the probability of selection [29]. We restricted the data to the nine countries, all in Southern Africa, with HIV prevalence rates in excess of 10% [30] to focus on the epicentre of the epidemic. Within these nine countries, SACMEQ III surveyed 39,664 grade six students from 1,807 schools.

Two different measures of HIV-related discrimination towards children living with HIV were used. The first from the question: “*A close friend of yours has told you that he or she is infected with HIV. How would you behave towards him or her?*” with the options being “*1) I would be more friendly than before; 2) I would behave the same as before, 3) I would avoid or shun him/her, 4) I am not sure how I would behave*”. The second from the question: “*Should a pupil who is infected with HIV be allowed to continue to attend school?*” with the available options being *“No”, “Yes”* and *“I am not sure.”* For our regression analysis we created a binary variable ( = 1) denoting students who reported they would avoid or shun a friend with HIV compared to those who would behave the same or be more friendly ( = 0). Similarly, we created a binary variable ( = 1) for students who believed that an HIV-positive student should *not* be allowed to attend school compared to children who believed they should be allowed. As both of these main dependent variables excluded individuals who reported being “unsure” we also created ordinal variables that included the “unsure” responses for the purposes of sensitivity analysis. For these variables, a non-discriminatory response was coded as the base category (0), unsure as the middle value (1) and discriminatory responses = 2.

### Analysis

We first computed descriptive statistics for our main outcome measures by country. Levels of discrimination are also presented by urban vs rural, poorest vs richest quartiles (index of household assets) and gender, as previous studies have found variation in HIV-related stigma and discrimination by geographic location [24], [31], socioeconomic status [32], and gender [22]. We then utilised multivariate logistic regression analysis to assess factors associated with our two binary measures of discrimination. In addition to geographic location (rural vs urban) and gender, several other explanatory variables were created and included in the models. Children's understanding of illness causation, contamination and contagion will differ at different stages of development [33]. HIV-related stigma and discrimination may therefore vary with age as beliefs about HIV and how it is transmitted change as children grow up. While the majority of the SACMEQ sample was between the ages of 12 and 14, the age range of students was wide, with 15% younger than 12 and eight percent older than 15. A continuous measure of age was accordingly included in our models to assess whether there was an association between age and discrimination. A measure of household wealth was formed using 31 questions regarding asset ownership. To create the index we used the first component from a principal component analysis of the 31 assets for each country. The average household asset score within each school was used as a proxy for the wealth level of the school. A binary variable was formed to indicate whether the student was an orphan (at least one deceased parent) as experiences of discrimination and bullying are often affected by orphan status [4] and these experiences could influence discrimination towards others. We also included a measure of mothers' education to control for intergenerational education and learning effects. Education, for example, is often negatively correlated with levels of stigma and discriminatory attitudes [21], [32]. Children of more educated mothers may therefore have grown up in a less stigmatising environment. We created dummy variables to compare students whose mother had less than secondary schooling to those whose mother had completed some secondary schooling and those whose mother had completed more than secondary school. Given the large percentage of “*don't know*” responses (12%) to mother's education we included an indicator of those who did not know their mother's education to avoid a substantial reduction in sample size.

As general levels of education and HIV knowledge have been found to be negatively associated with stigma [21] we first included the SACMEQ reading score (range: 63–966) as a measure of ability/intelligence. The reading score was created by SACMEQ using standard Rasch scaling procedures on the 55 reading questions [34]. We then formed a measure of general HIV knowledge based on five true or false questions on transmission knowledge and five on HIV prevention knowledge drawn from the SACMEQ HIV knowledge test. Questions were selected if they measured transmission or prevention knowledge and the underlying construct had not been measured by a previous question (according to independent assessment by both authors). The transmission knowledge questions were (1) “*A person can get HIV the first time he or she has unprotected sex with a person who has HIV*”, (2) “*A person can get HIV by having sex once without using a condom*”, (3) “*HIV can be transmitted by vaginal fluids*”, (4) “*HIV can be transmitted by semen/sperm*”, (5) “*Having more than one sexual partner could increase the risk of getting HIV*”. The HIV prevention questions included (1) “*One way to prevent getting HIV is abstaining from sex*”, (2) “*Having sex only with people who look healthy is one way to prevent getting HIV*”, (3) “Delaying starting to have sex is one way to reduce the risk of getting HIV”, (4) “*The correct use of condoms offers protection against HIV*”, (5) “*A person can be protected from getting HIV by having only one sex partner who is not infected and also has no other sex partners*.” Correct responses to these questions were summed and divided by the number of questions for which data was given (minimum of 50% required) so the HIV knowledge measure represents the proportion of correct responses. We also included an index which was formed as a count of the number of sources (0–23) from which students reported receiving information about HIV and AIDS, such as radio, TV, and internet (see Digital Content S1 for a full list of potential sources of information included in the survey).

Negative attitudes towards people living with HIV that are driven by fears of contracting HIV via casual contact (often referred to as instrumental stigma) have been shown to be a powerful driver of discrimination (or enacted stigma) among adults [35]. Accordingly, to test this relationship among children, we formed a measure of the degree to which students believed that HIV could be contracted via casual contact using eight questions from the HIV test. The following true and false questions were used: (1) “*A person can get HIV from mosquito bites*”, (2) “*A person can get HIV by sitting next to a person who has AIDS*”, (3) “*A person can get HIV by swimming in the same water as a person who has AIDS*”, (4) “*A person can get HIV by sitting on a toilet seat that has been used by a person who has AIDS*”, (5) “*A person can get HIV by sharing food with a person who has AIDS*”, (6) “*A person can get HIV by hugging a person who has AIDS*”, (7) “*A person can get HIV by wearing clothes used by a person who has AIDS*”, (8) “*A person can spread HIV by coughing*.” Similar to the creation of the HIV knowledge measure, correct responses to these questions were summed and divided by the number of questions for which data was given (minimum of 50% required) so the casual contact measure represents the proportion of responses for which students indicated that HIV could be spread via casual contact.

Qualitative research in Zambia concluded that adults often believe that individuals living with HIV are bewitched [36]. The findings of this qualitative research indicated that this belief may transfer blame away from sick individuals and to the bewitcher, and consequently, reduce stigma toward HIV positive individuals. On the other hand, ethnographic research conducted in Botswana found that instead of decreasing blame and stigma, occult explanations of HIV infection furthered the social marginalisation of HIV-infected children and their family [37]. Quantitative research, also from Botswana, found that women who believed that a person could get HIV through witchcraft were more likely to express discriminatory attitudes towards a teacher with HIV [21]. The relationship between HIV-related stigma and discrimination and beliefs about a link between witchcraft and HIV is, therefore, unclear. To assess this relationship among students we included a binary variable to identify students who held such beliefs as measured by the question “*A person with AIDS is bewitched.*” Finally, previous studies have found that contact with people living with HIV has been associated with greater acceptance of people living with HIV [38]. Accordingly, we created a binary variable to identify students who had met someone living with HIV as measured by whether the student reported receiving information about HIV from an HIV positive individual.

All analyses were conducted with Stata 12.0 (Stata Corporation, College Station, Texas, United States of America). All standard errors have been adjusted to account for the two-stage survey design with stratification by province and clustering by school.

## Results

Table 1 presents summary statistics of sample characteristics, including knowledge and beliefs about HIV. The sample of 39,664 students comprised roughly equal proportions of girls and boys, with a median age range across countries of between 12 and 14 years old. There was a high degree of variation in geographic location across countries (as reported by the School Head) with the proportion from rural areas being 37% in Mozambique and 70% and greater in Malawi, Swaziland and Zimbabwe. More than one in four students in all countries reported the loss of at least one parent. In terms of HIV information and knowledge, students reported receiving information about HIV from a variety of sources (averages between 10 sources in Lesotho and 13.5 sources in Botswana and Swaziland). The most common cited sources of information were classes at school, radio, clinic, magazines/newspapers and books. On average, respondents answered 71% of the HIV knowledge questions correctly with a range from around 67% in Lesotho and Zimbabwe, to 75% in Swaziland. Respondents scored lowest on the question “*Having sex only with people who look healthy is one way to prevent getting HIV*”, with around half or more answering true to this statement in Lesotho, Mozambique, Namibia, South Africa and Zambia. The vast majority (72%) reported that HIV could be transmitted via some form of casual contact, with the average respondent reporting that transmission was possible in two of the eight scenarios. Beliefs that HIV could be spread via casual contact were most widespread in Lesotho, where respondents, on average, reported in 35% of the questions that casual contact was possible, and least common in Swaziland. More than a quarter of respondents in all countries and around 40% in Botswana, Lesotho, South Africa and Zimbabwe, answered true to the statement that a person can spread HIV by coughing. Finally, Table 1 shows that a significant minority of students in all countries had received information about HIV from someone living with HIV.

**Table 1:** Sample characteristics.

	Botswana	Lesotho	Malawi	Mozambique	Namibia	South Africa	Swaziland	Zambia	Zimbabwe	Total
**Number of schools**	160	182	139	183	267	392	172	157	155	1807
**Sample size (Gr6 students)**	3868	4240	2781	3360	6398	9071	4030	2895	3021	39664
**Median age (years)**	12.6	13.8	14.0	13.9	13.3	12.6	13.6	13.9	12.3	13.5
**Interquartile range of age (years)**	1.3	2.4	2.3	2.5	2.2	1.3	2.5	2.4	1.3	2.1
**Percentage girls**	50%	55%	49%	46%	52%	51%	50%	49%	56%	51%
**Percentage rural**	48%	66%	76%	37%	61%	50%	70%	65%	71%	59%
**HIV prevalence among 15–49 year olds (UNAIDS, 2012)**	23%	23%	11%	11%	13%	18%	27%	13%	15%	NA
**Orphan status (either parent deceased)**	31%	40%	28%	29%	33%	31%	41%	29%	36%	33%
**(Std. Err)**	1.4%	1.0%	1.0%	0.9%	1.3%	1.2%	1.0%	1.1%	1.3%	0.4%
**Sources of HIV information (mean of 0–23 index)**	13.5	10.2	13.1	10.8	13.0	13.0	13.7	11.9	11.9	12.487
**(Std. Err.)**	0.18	0.19	0.27	0.18	0.14	0.14	0.17	0.30	0.27	0.06
**General HIV knowledge** a	71%	68%	72%	72%	72%	72%	75%	70%	68%	71%
**(Std. Err.)**	0.6%	0.5%	0.9%	0.7%	0.5%	0.5%	0.5%	0.7%	0.7%	0.2%
**Casual Contact beliefs** b	26%	35%	24%	24%	22%	22%	15%	26%	25%	24%
**(Std. Err.)**	0.8%	1.0%	0.9%	0.7%	0.6%	0.6%	0.5%	0.9%	1.0%	0.3%
**Beliefs that people with AIDS are bewitched** c	21%	32%	17%	37%	25%	21%	25%	22%	24%	25%
**(Std. Err.)**	1.0%	1.4%	1.2%	1.4%	1.0%	0.9%	1.3%	1.3%	1.5%	0.4%
**Interaction with people living with HIV**	45%	29%	34%	30%	41%	38%	36%	44%	42%	38%
**(Std. Err.)**	1.9%	1.6%	2.3%	1.2%	1.6%	1.4%	2.4%	2.3%	2.2%	0.6%

Notes:

a The figures for the general HIV knowledge measure represent the average proportion of the 10 relevant true-or-false statements for which students reported the correct response.

b The figures for the casual contact beliefs measure represent the average proportion of the eight relevant true-or-false statements for which students indicated that HIV could be spread via casual contact.

c The ‘beliefs that people with AIDS are bewitched’ measure represents the proportion of students who responded “true” to the statement “A person with AIDS is bewitched.”

Table 2 displays levels of discrimination in each country. Levels of discrimination varied significantly between the nine countries. While only one in ten grade six students in Botswana, Malawi, South Africa and Swaziland would “avoid or shun” a close friend who was HIV positive, the proportions in Lesotho, Mozambique, Zambia and Zimbabwe were twice as high (20%). As expected, the percentage of children who believed that HIV positive students should not be allowed to continue to attend school was, on average, considerably higher than the proportion that would avoid or shun a close friend who was HIV positive, the latter being the more severe manifestation of stigma. Of note, large proportions of students believed that HIV positive children should not be allowed to continue to attend school, particularly in Zambia (33%), Lesotho (37%) and Zimbabwe (42%). The corresponding figures for Malawi and Swaziland were considerably lower at 13% and 12% respectively. See Digital Content S2 and S3, for exact percentages and standard errors for each of the response options for our two questions on discrimination by country, gender, rural vs urban and richest vs poorest quartiles.

**Table 2:** Levels of HIV discrimination by country.

Country	Proportion of Grade 6 students who would “avoid or shun” a close friend who revealed that they were HIV+	*Standard error*	Proportion of Grade 6 students who believe that HIV+ students should not be allowed to continue to attend school	*Standard error*
Botswana	10.6%	*0.8%*	26.4%	*1.3%*
Lesotho	23.4%	*1.6%*	37.2%	*2.0%*
Malawi	8.2%	*0.9%*	12.1%	*1.4%*
Mozambique	20.4%	*1.3%*	25.2%	*1.2%*
Namibia	13.9%	*0.8%*	25.8%	*1.3%*
South Africa	8.9%	*0.6%*	21.7%	*1.0%*
Swaziland	11.9%	*1.1%*	12.9%	*1.0%*
Zambia	19.8%	*1.2%*	33.4%	*1.8%*
Zimbabwe	20.3%	*1.3%*	41.7%	*2.1%*

**Figure 1:**
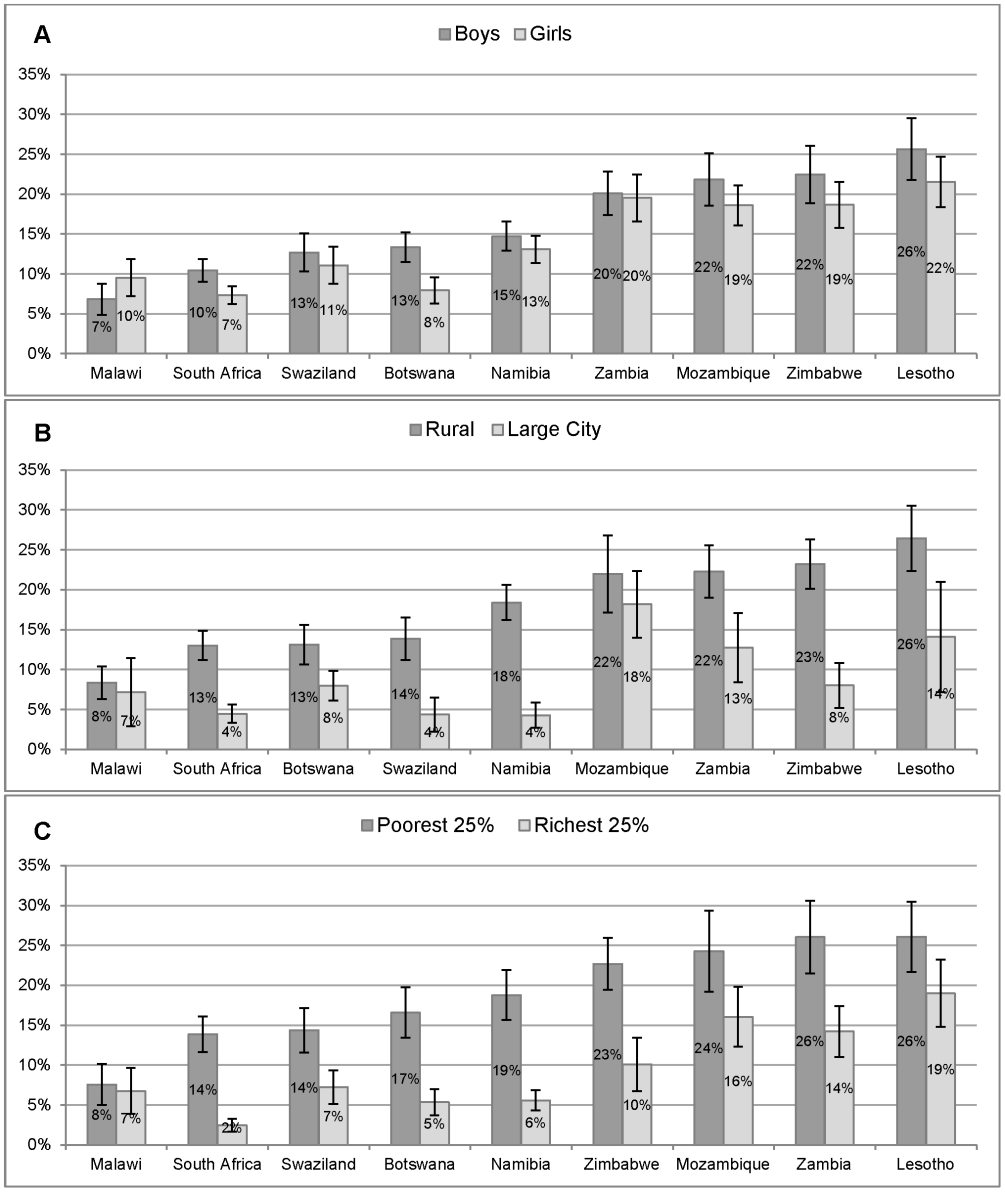
Response to an HIV-positive status of a close friend. Proportion of grade 6 students that would “avoid or shun” a close friend who revealed that they were living with HIV by gender (Figure 1A), geographic location (Figure 1B) and wealth quartile (Figure 1C) including 95% confidence interval).

Figure 1 illustrates the differences in proportions who would shun a close friend with HIV by gender (Figure 1A), by those attending schools situated in rural areas compared to large cities (Figure 1B), and between the richest and poorest 25% of students (Figure 1C). Generally, differences between boys and girls were small with slightly more boys than girls who reported discrimination, although these differences are only statistically significant in Botswana and South Africa. Much larger differences were found by geographic location and socioeconomic status. In most countries, significantly greater proportions of students living in rural areas compared to urban areas and poorer students compared to richer students reported discrimination. In Namibia, South Africa and Swaziland, discrimination appeared to be concentrated in the rural areas and among the poor with relatively few students in urban areas and in the top socioeconomic quartile in these countries reporting discrimination.

**Figure 2:**
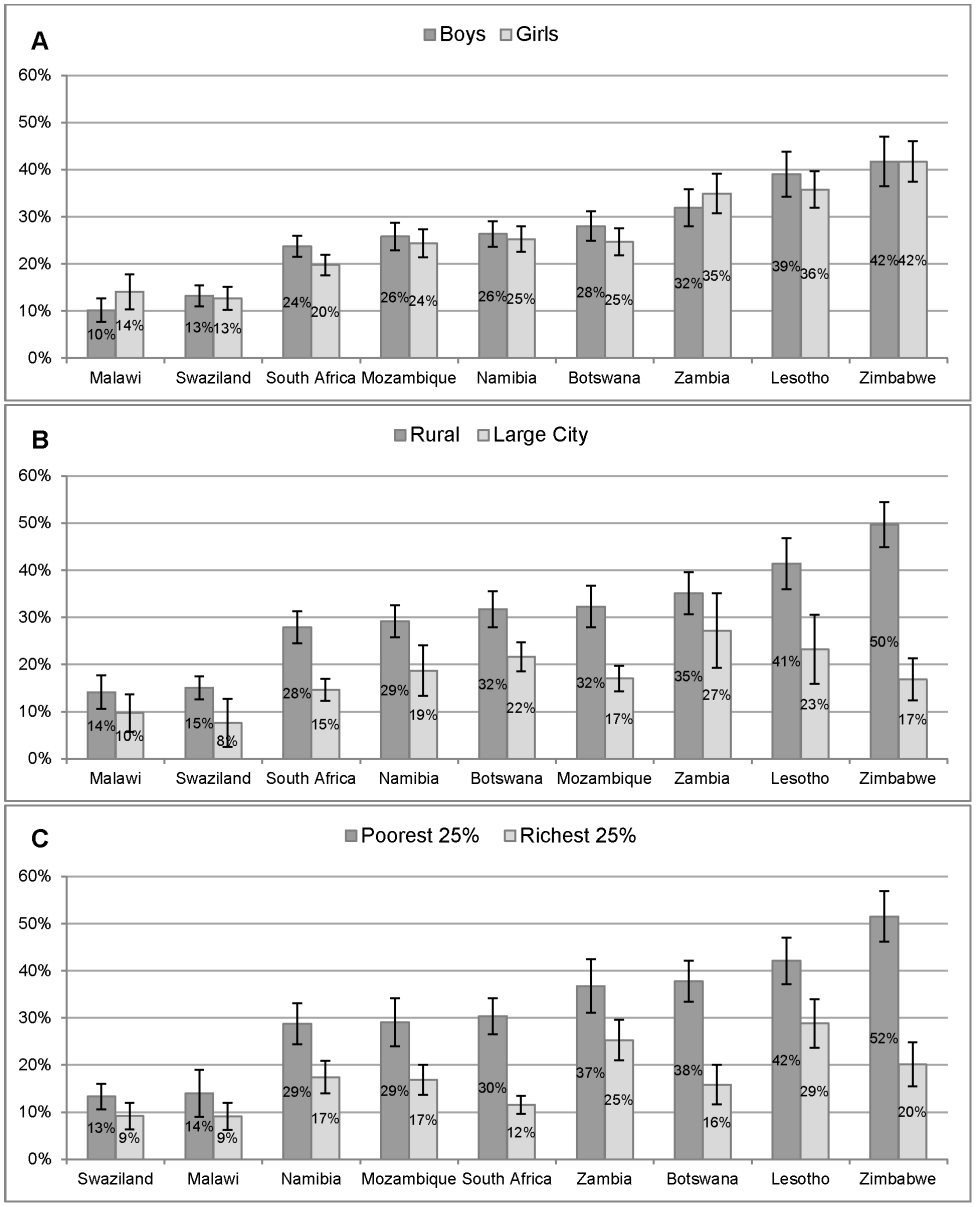
Beliefs that a student living with HIV should not be allowed to attend school. Proportion of grade 6 students who believed that students living with HIV should not be allowed to continue to attend school, by gender (Figure 2A), geographic location (Figure 2B) and wealth quartile (Figure 2C) including 95% confidence interval.

In Figure 2 we see similar patterns in the proportions who reported that an HIV-positive student should not be allowed to attend school by gender (Figure 2A), geographic location (Figure 2B) and socioeconomic quartile (Figure 2C) as Figure 1 displayed. These figures emphasised variation in discrimination by subpopulations across the region. In Figure 2B, for example, results show that 8% of the urban sample in Swaziland reported that an HIV-positive student should not be allowed to attend school compared to 50% in the rural sample from Zimbabwe.

Table 3 presents the results from the multivariate regression models for our binary dependent variable of whether students believed they would avoid or shun a close friend with HIV. Variation was evident in both the factors influencing discrimination in each country and the direction of the relationship between these factors and discrimination. Controlling for other factors, girls were less likely to report discrimination in Botswana (OR = 0.70; p<0.01), Lesotho (OR = 0.82; p<0.05) and South Africa (OR = 0.76; p<0.01), but more likely in Malawi (OR = 1.3; p<0.1). The direction of the association between age and discrimination (as measured by whether students believed they would avoid or shun a close friend with HIV) varied between countries, but the relationship was not statistically significant in any country. Mixed results were also found for the effect of orphan status, mother's education and having met a person living with HIV. Discrimination was less likely to be reported by students from rural areas in Zambia as compared to their urban counterparts. In Mozambique, Namibia, South Africa and Swaziland students attending more affluent schools were less likely to report discrimination than their peers in poorer schools. In Botswana, Malawi and South Africa, exposure to a greater number of sources of HIV information (and in South Africa, greater levels of general HIV knowledge) was associated with lower odds of reporting discrimination. The factors with the most consistent relationship with discrimination across all countries were reading ability and beliefs about HIV transmission via casual contact. In all countries, except Mozambique, increased reading ability was associated with lower levels of reporting discrimination and in all countries except Swaziland there was a strong positive association between beliefs about HIV being spread via casual contact and increased reported discrimination with the largest effect found for students in Malawi.

**Table 3:** Logistic regression (adjusted odds ratios presented) for beliefs that students would shun or avoid a close friend with HIV.

	Botswana	Lesotho	Malawi	Mozambique	Namibia	South Africa	Swaziland	Zambia	Zimbabwe
Girl ( = 1)	0.7001***	0.8178**	1.3212*	0.9295	0.8916	0.7560***	0.995	0.9796	0.8751
Age	0.9996	0.9771	0.9674	1.0035	0.9584	1.0235	1.0262	1.0076	1.0041
Rural ( = 1)	1.1499	1.2153	1.3778	0.8284	1.2614	1.1384	1.256	1.4928*	1.0914
Reading score	0.9940***	0.9948***	0.9956**	1.0051***	0.9956***	0.9953***	0.9927***	0.9957***	0.9952***
Orphan	0.9394	1.1049	0.7505*	1.023	1.1778*	0.9934	1.1008	0.8256*	0.9195
Mother's education									
Some secondary	0.9858	0.939	1.1453	0.5078***	0.7986*	0.9031	0.9242	0.8954	1.1727
>secondary	1.0632	0.8425	2.0884**	0.9894	0.8564	0.6519***	1.1695	1.175	0.9754
Don't know	0.9899	1.2148	0.844	0.8372	1.1779	0.9791	0.7306**	0.944	0.9669
Exposure to HIV info	0.9324***	0.98	0.9476*	1.021	0.9761	0.9543***	0.9953	0.9722	1.0223
HIV knowledge	0.6569	0.8986	1.3074	0.9458	0.8158	0.5818**	0.7293	1.1062	1.2575
HIV spread via casual contact	1.6926**	2.6182***	4.9743***	1.5085*	2.3660***	2.6097***	1.1364	2.8364***	2.4520**
Bewitched	1.0038	0.9404	1.1845	1.1754	1.0257	0.9081	0.9572	1.0549	1.082
Have met person living with HIV	1.233	0.7969*	2.1227***	1.1303	0.9441	1.2063	1.0641	1.3569**	0.5821***
Socioeconomic status (SES)	0.97	1.0012	0.9749	0.978	1.0136	1.0142	0.9906	1.0053	1.0028
School SES	0.9934	0.9795	1.094	0.8339***	0.8965*	0.8371***	0.7851**	1.0297	0.9265
Constant	11.0646**	7.4437**	0.5612	0.0243***	4.5249**	1.9227	8.5990**	1.3231	2.5324
N	2705	2861	2514	2419	4017	6068	2660	2193	2058

Notes:

* p<0.05,

** p<0.01,

*** p<0.001.

Robust standard errors, stratification by province, clustered at the school level.

Table 4 presents the results from the multivariate regression models for our binary dependent variable of whether participants believed that an HIV-positive student should not be allowed to attend school. Consistent with the results above, the models in Table 4, show that factors significantly associated with discrimination varied by country. But, in contrast to the previous models, the factors that were significantly associated with discrimination tended to have a similar direction of association. The relationship between gender and discrimination was only significant in South Africa with girls less likely to report that a student living with HIV should not be allowed to attend school (OR = 0.88, p<0.1). The relationship between age and the belief that an HIV-positive student should not be allowed to attend school was significant in Namibia (OR = 0.94, p<0.05), South Africa (OR = 0.95, p<0.1) and Swaziland (OR = 0.94, p<0.05), with older individuals less likely to report that a student living with HIV should not be allowed to attend school. Additional analysis (available upon request) indicated little difference between the two younger age groups (10–12 years old vs 12–14 years old) in these countries, and that the association by age was driven by lower levels of reported discrimination among students 15 years and older. In Malawi (OR = 3.1), Mozambique (OR = 1.7) and Swaziland (OR = 2.4) students in rural areas were significantly (p<0.01) more likely to report discrimination than students in urban areas. For students in Namibia, South Africa and Zambia, there was a negative association between mother's education and levels of discrimination. Generally, there was a negative association between discrimination and reading ability, exposure to HIV information and general HIV knowledge. No statistically significant relationship was found between discrimination and beliefs that a person with AIDS is bewitched. Finally, consistent with the previous models, the greater the misconception that HIV can be transmitted via casual contact the greater the odds of students reporting discrimination. Sensitivity analysis showed that results are robust to the inclusion of the “don't know” responses as a middle category in the dependent variable (ordinal logistic regression models; results available upon request).

**Table 4:** Logistic regression (adjusted odds ratios presented) for beliefs that an HIV positive student should not be allowed to attend school.

	Botswana	Lesotho	Malawi	Mozambique	Namibia	South Africa	Swaziland	Zambia	Zimbabwe
Girl ( = 1)	1.0102	0.949	1.2371	0.9343	1.0316	0.8820*	0.9536	1.1127	1.0915
Age	0.9633	0.9722	0.9939	1.0013	0.9375**	0.9467*	0.9360**	0.9859	0.9794
Rural ( = 1)	1.1929	1.1555	3.141***	1.6961***	0.7918	1.1619	2.3931***	1.0683	1.6479
Reading score	0.9960***	0.9939***	0.993***	0.9976***	0.9952***	0.9965***	0.9945***	0.995***	0.996***
Orphan	1.0463	1.0818	0.9085	1.1412	0.9595	1.0399	0.9489	0.8754	0.9364
Mother's education									
Some secondary	0.9963	0.9233	1.2407	1.0451	0.9348	0.9345	0.8533	0.7312**	1.0081
>secondary	0.9652	0.8872	0.7479	0.9661	0.8066*	0.7744**	0.9169	0.8064	0.7404
Don't know	1.0199	1.098	0.9717	1.0963	1.3082**	1.0186	1.054	0.6988*	0.9349
Exposure to HIV info	0.8971***	0.9628*	0.9367**	0.9715*	0.9336***	0.9341***	0.9449**	0.9799	0.9450***
HIV knowledge	0.2085***	0.5982*	1.1859	0.5771*	0.3814***	0.5327***	0.4539**	0.6126*	0.4689**
HIV spread via casual contact	4.4772***	4.6884***	7.080***	2.2830***	5.3768***	3.9044***	3.1241***	2.679***	2.6114***
Bewitched	1.0739	0.9026	0.9217	1.0252	1.1675	1.0156	1.1135	0.9513	0.83
Have met person living with HIV	1.0605	0.8176	1.2487	1.1148	0.9425	1.2718*	1.1008	1.0709	1.1049
Socioeconomic status (SES)	0.9884	0.9872	1.0091	0.9998	1.024	1.0192	1.0127	0.9891	1.0496
School SES	1.0396	1.111	1.1387	1.0446	0.9964	0.9409	1.1944	1.0443	0.8882
Constant	53.437***	25.794***	1.4043	1.9946	48.106***	10.2768***	15.240***	9.576***	12.189**
N	2947	3443	2673	2362	4897	7085	3403	2509	2471

Notes:

* p<0.05,

** p<0.01,

*** p<0.001.

Robust standard errors, stratification by province, clustered at the school level.

## Discussion

This study contributes to an emerging literature on child-on-child HIV-related discrimination by examining levels of discrimination and associated factors among a large sample of grade six students across nine countries in southern Africa. Results showed that levels of discrimination were high in particular sub-groups across the region and varied significantly between countries, all of which supports previous research on stigma and discrimination [24], [31] as well as more recent qualitative findings indicating that stigma and discrimination of children by children may be relatively common [23]. Similarly, factors associated with discrimination varied widely between countries. More than one in five students from rural areas in Mozambique, Zambia, Zimbabwe and Lesotho reported that they would shun or avoid a close friend if that friend disclosed an HIV-positive status. Furthermore, in all countries except Malawi and Swaziland, more than one in four students from rural areas reported the belief that an HIV-positive student should not be allowed to attend school.

The implications of these results should be considered in conjunction with the limitations of this study. Mainly, that the SACMEQ questionnaire was not designed specifically to assess factors associated with discrimination and thus there is the potential for unobserved confounding factors. Given that statements relating to HIV knowledge and beliefs were structured in true-or-false format, and without a “don't know” response option, student guessing may affect measures created from these statements. Furthermore, we did not have a direct measure of stigmatising attitudes (i.e. negative moral judgement) or actual discriminatory behaviours towards children living with HIV and therefore we do not know to what degree the reported preferences for social distance are associated with these factors among children. In addition, the study sample frame was grade six students currently attending school and therefore our results are not generalisable to more marginalised populations who were not attending school. The experience of, and propensity to stigmatise may differ significantly between more marginalised children (i.e. the un-enrolled) than those attending school. It would therefore be informative for future research on stigma among children to include these marginalised populations.

Our findings reiterate that stigmatisation and discrimination are complex social processes, which are clearly influenced by local context, and therefore vary widely by country, school location and socioeconomic status. Results from one setting may not translate to neighbouring areas and this highlights the importance of context specific research and programming. Previous research found that stigma among school students could be gendered, with boys in Swaziland reporting greater stigmatising attitudes than girls [22]. Our study found relatively small differences in HIV-related discrimination between girls and boys, and although in most countries girls reported slightly less discrimination, in Malawi the opposite was true. Therefore, our evidence does not point towards the need for gender specific interventions. Overall, our study also found relatively small differences in HIV-related discrimination by age, which suggests that interventions aimed at grade 6 students also do not need to be tailored to different age groups. In a few countries there was some evidence that older children may be less discriminatory. It would be informative for future research to assess changes in HIV-related stigma and discrimination as children progress through school and the underlying mechanisms of change.

One of the strengths of the multi-country nature of our study is that we could determine factors that influence HIV-related discrimination that traverse different contexts. Findings show that beliefs that HIV can be transmitted via casual contact could be a strong factor that influences discriminatory outcomes across the region. Our results indicate that reductions in HIV-related discrimination among children may be achieved through interventions to correct these misconceptions around casual contact. Notwithstanding the above, we are also aware that improving knowledge and beliefs regarding the spread of HIV via casual contact with people living with HIV will not be sufficient to eliminate HIV-related discrimination. We did not find evidence that children who believed that a person with AIDS is bewitched would be less discriminating towards a child with HIV. This suggests that the hypothesis of such a relationship among adults [36] may not extend to young children. However, further research should assess the relationship between beliefs about witchcraft and stigmatising attitudes among children, especially given the substantial proportion of children in our study who reported the belief that a person with AIDS is bewitched.

Overall, our study points toward the need for early interventions (grade six or before) to reduce stigma and discrimination among children, especially in schools in rural and poorer areas. In particular, interventions should aim to correct misconceptions that HIV can be transmitted via casual contact with people living with HIV.

## Supporting Information

Digital Content S1 List of sources from which students reported receiving information about HIV and AIDS.
(DOCX)

Digital Content S2 Table: Student responses to “A close friend of yours has told you that he or she is infected with HIV. How would you behave towards him/her?” by sub-groups.
(DOCX)

Digital Content S3 Table: Student responses to “Should a pupil who is infected with HIV be allowed to continue to attend school?” by sub-groups.
(DOCX)
