# Supplementary material for: HIV-Related Discrimination among Grade Six Students in Nine Southern African Countries
Source: PLoS One. 2014 Aug 8;9(8):e102981. doi: 10.1371/journal.pone.0102981 (PMC4126685; doi:10.1371/journal.pone.0102981)
Supplement: Digital Content S1 — List of sources from which students reported receiving information about HIV and AIDS. (DOCX) [file pone.0102981.s001.docx]

**Digital content S1:** List of sources from which students reported receiving information about HIV and AIDS.

1. Radio
2. TV
3. Video player (VCR, DVD, etc)
4. Internet
5. Computer(s)
6. Poster(s) / Billboard(s)
7. Book(s)
8. Magazine(s)/Newspaper(s)
9. Drama(s)/Play(s)/Concert(s)
10. School club
11. Cinema (in-door, out-door, or mobile)
12. Recreational activities
13. Classroom lesson(s)
14. Hospital / Clinic
15. Teacher(s) / School Head
16. Friend(s)
17. Counsellor(s)
18. Peer educator(s)
19. Doctor(s)
20. Community health worker(s)
21. Person(s) from church, mosque, temple, etc.
22. A person living with HIV
23. Family / Relatives
