# Supplementary material for: HIV-Related Discrimination among Grade Six Students in Nine Southern African Countries
Source: PLoS One. 2014 Aug 8;9(8):e102981. doi: 10.1371/journal.pone.0102981 (PMC4126685; doi:10.1371/journal.pone.0102981)
Supplement: Digital Content S2 — Table: Student responses to “A close friend of yours has told you that he or she is infected with HIV. How would you behave towards him/her?” by sub-groups. (DOCX) [file pone.0102981.s002.docx]

Digital content S2, Table: Student responses to "A close friend of yours has told you that he or she is infected with HIV. How would you behave towards him/her?" by sub-groups.

|  |  | **National** | *SE* | **Rural** | *SE* | **Small Town** | *SE* | **Large City** | *SE* | **Poorest 25%** | *SE* | **Richest 25%** | *SE* | **Boys** | *SE* | **Girls** | *SE* |
| --- | --- | --- | --- | --- | --- | --- | --- | --- | --- | --- | --- | --- | --- | --- | --- | --- | --- |
| Botswana | More friendly | 30.8% | *1.4%* | 26.6% | *1.8%* | 34.1% | *3.8%* | 35.1% | *2.0%* | 23.6% | *1.9%* | 38.3% | *1.9%* | 28.6% | *1.6%* | 32.9% | *1.5%* |
|  | Same | 28.3% | *1.2%* | 29.3% | *1.7%* | 29.7% | *2.7%* | 25.7% | *1.9%* | 28.0% | *1.9%* | 28.7% | *2.3%* | 28.5% | *1.4%* | 28.2% | *1.4%* |
|  | Avoid/Shun | 10.6% | *0.8%* | 13.1% | *1.3%* | 8.8% | *1.4%* | 8.0% | *1.0%* | 16.6% | *1.6%* | 5.3% | *0.8%* | 13.4% | *1.0%* | 7.9% | *0.8%* |
|  | Not sure | 30.3% | *1.3%* | 30.9% | *2.0%* | 27.4% | *3.0%* | 31.2% | *2.0%* | 31.8% | *2.1%* | 27.6% | *2.3%* | 29.6% | *1.5%* | 30.9% | *1.6%* |
| Lesotho | More friendly | 20.8% | *1.2%* | 18.5% | *1.4%* | 21.1% | *2.4%* | 30.0% | *3.2%* | 17.2% | *1.5%* | 26.8% | *1.9%* | 18.8% | *1.3%* | 22.4% | *1.4%* |
|  | Same | 25.3% | *1.1%* | 25.7% | *1.6%* | 24.7% | *1.8%* | 24.3% | *1.9%* | 25.0% | *1.9%* | 24.1% | *1.7%* | 25.5% | *1.4%* | 25.0% | *1.3%* |
|  | Avoid/Shun | 23.4% | *1.6%* | 26.5% | *2.1%* | 20.4% | *3.0%* | 14.1% | *3.5%* | 26.1% | *2.2%* | 19.0% | *2.2%* | 25.6% | *2.0%* | 21.5% | *1.6%* |
|  | Not sure | 30.6% | *1.3%* | 29.4% | *1.5%* | 33.9% | *2.8%* | 31.7% | *3.6%* | 31.7% | *2.0%* | 30.1% | *2.0%* | 30.0% | *1.5%* | 31.0% | *1.5%* |
| Malawi | More friendly | 35.6% | *2.2%* | 35.6% | *2.5%* | 36.7% | *7.6%* | 34.7% | *5.4%* | 36.4% | *3.6%* | 36.8% | *3.0%* | 36.7% | *2.2%* | 34.5% | *2.6%* |
|  | Same | 46.6% | *2.1%* | 45.7% | *2.3%* | 49.0% | *8.1%* | 49.6% | *5.5%* | 44.9% | *3.3%* | 46.6% | *3.0%* | 48.0% | *2.2%* | 45.1% | *2.6%* |
|  | Avoid/Shun | 8.2% | *0.9%* | 8.4% | *1.0%* | 7.9% | *3.6%* | 7.2% | *2.2%* | 7.6% | *1.3%* | 6.7% | *1.5%* | 6.8% | *1.0%* | 9.5% | *1.2%* |
|  | Not sure | 9.6% | *1.0%* | 10.3% | *1.3%* | 6.3% | *1.9%* | 8.5% | *2.0%* | 11.1% | *1.9%* | 9.8% | *1.5%* | 8.4% | *1.0%* | 10.9% | *1.4%* |
| Mozambiq. | More friendly | 24.1% | *1.1%* | 23.0% | *1.6%* | 23.3% | *2.4%* | 25.8% | *1.9%* | 21.3% | *1.7%* | 28.6% | *2.2%* | 25.3% | *1.3%* | 22.7% | *1.4%* |
|  | Same | 30.4% | *1.2%* | 31.9% | *2.0%* | 33.7% | *3.0%* | 26.5% | *1.7%* | 33.2% | *2.7%* | 23.5% | *1.9%* | 29.5% | *1.6%* | 31.5% | *1.5%* |
|  | Avoid/Shun | 20.4% | *1.3%* | 22.0% | *2.5%* | 21.2% | *2.2%* | 18.2% | *2.1%* | 24.3% | *2.6%* | 16.1% | *1.9%* | 21.8% | *1.7%* | 18.6% | *1.3%* |
|  | Not sure | 25.1% | *1.1%* | 23.1% | *1.8%* | 21.8% | *1.8%* | 29.5% | *2.2%* | 21.3% | *1.9%* | 31.9% | *2.4%* | 23.4% | *1.4%* | 27.2% | *1.3%* |
| Namibia | More friendly | 25.2% | *1.0%* | 21.3% | *1.2%* | 26.3% | *2.2%* | 36.3% | *2.0%* | 18.6% | *1.5%* | 34.0% | *1.6%* | 23.9% | *1.0%* | 26.4% | *1.3%* |
|  | Same | 24.7% | *0.9%* | 27.7% | *1.3%* | 21.4% | *1.4%* | 18.6% | *1.6%* | 29.8% | *1.8%* | 22.0% | *1.3%* | 24.7% | *1.1%* | 24.7% | *1.1%* |
|  | Avoid/Shun | 13.9% | *0.8%* | 18.4% | *1.1%* | 9.3% | *1.3%* | 4.3% | *0.8%* | 18.8% | *1.6%* | 5.6% | *0.6%* | 14.7% | *0.9%* | 13.1% | *0.9%* |
|  | Not sure | 36.3% | *1.1%* | 32.6% | *1.4%* | 43.0% | *2.7%* | 40.9% | *1.9%* | 32.9% | *1.9%* | 38.5% | *1.5%* | 36.7% | *1.2%* | 35.9% | *1.4%* |
| South Africa | More friendly | 32.2% | *1.2%* | 27.4% | *1.7%* | 38.3% | *2.7%* | 36.2% | *1.8%* | 26.0% | *2.0%* | 37.1% | *1.6%* | 29.7% | *1.2%* | 34.6% | *1.4%* |
|  | Same | 27.7% | *1.0%* | 29.5% | *1.6%* | 23.7% | *1.9%* | 27.1% | *1.5%* | 26.0% | *1.6%* | 31.4% | *1.5%* | 27.8% | *1.1%* | 27.5% | *1.1%* |
|  | Avoid/Shun | 8.9% | *0.6%* | 13.0% | *0.9%* | 5.1% | *0.7%* | 4.5% | *0.6%* | 13.9% | *1.1%* | 2.4% | *0.4%* | 10.4% | *0.7%* | 7.3% | *0.6%* |
|  | Not sure | 31.3% | *1.1%* | 30.1% | *1.5%* | 32.9% | *2.6%* | 32.3% | *2.0%* | 34.2% | *1.9%* | 29.1% | *1.5%* | 32.1% | *1.1%* | 30.5% | *1.3%* |
| Swaziland | More friendly | 27.2% | *1.4%* | 25.1% | *1.7%* | 28.5% | *2.8%* | 35.2% | *3.7%* | 24.0% | *1.8%* | 34.2% | *2.1%* | 27.0% | *1.5%* | 27.3% | *1.7%* |
|  | Same | 27.0% | *1.2%* | 26.4% | *1.5%* | 30.8% | *2.5%* | 26.1% | *3.2%* | 26.4% | *1.9%* | 27.2% | *1.8%* | 27.5% | *1.4%* | 26.5% | *1.4%* |
|  | Avoid/Shun | 11.9% | *1.1%* | 13.9% | *1.4%* | 10.4% | *2.7%* | 4.4% | *1.1%* | 14.4% | *1.4%* | 7.2% | *1.1%* | 12.7% | *1.2%* | 11.1% | *1.2%* |
|  | Not sure | 34.0% | *1.5%* | 34.7% | *1.9%* | 30.3% | *2.5%* | 34.3% | *3.7%* | 35.3% | *2.1%* | 31.3% | *2.1%* | 32.8% | *1.8%* | 35.1% | *1.6%* |
| Zambia | More friendly | 22.9% | *1.4%* | 21.7% | *1.7%* | 25.8% | *3.1%* | 24.7% | *3.4%* | 20.0% | *2.5%* | 27.0% | *2.7%* | 24.2% | *1.7%* | 21.6% | *1.6%* |
|  | Same | 34.9% | *1.8%* | 35.4% | *2.2%* | 26.6% | *2.8%* | 39.6% | *5.3%* | 35.6% | *2.6%* | 32.7% | *3.2%* | 35.7% | *2.0%* | 34.1% | *2.0%* |
|  | Avoid/Shun | 19.8% | *1.2%* | 22.3% | *1.7%* | 18.5% | *2.7%* | 12.7% | *2.2%* | 26.0% | *2.3%* | 14.2% | *1.6%* | 20.1% | *1.4%* | 19.5% | *1.5%* |
|  | Not sure | 22.4% | *1.4%* | 20.6% | *1.7%* | 29.0% | *4.0%* | 23.0% | *3.3%* | 18.4% | *1.9%* | 26.0% | *2.5%* | 20.1% | *1.6%* | 24.8% | *1.7%* |
| Zimbabwe | More friendly | 23.1% | *1.3%* | 19.0% | *1.5%* | 28.0% | *4.2%* | 35.7% | *2.6%* | 19.1% | *2.4%* | 34.1% | *2.3%* | 22.0% | *1.7%* | 24.0% | *1.5%* |
|  | Same | 28.2% | *1.3%* | 26.8% | *1.6%* | 23.9% | *3.6%* | 33.5% | *2.3%* | 25.3% | *1.9%* | 30.4% | *2.2%* | 29.3% | *1.5%* | 27.3% | *1.7%* |
|  | Avoid/Shun | 20.3% | *1.3%* | 23.2% | *1.6%* | 23.6% | *4.9%* | 8.0% | *1.4%* | 22.7% | *1.7%* | 10.1% | *1.7%* | 22.5% | *1.8%* | 18.7% | *1.5%* |
|  | Not sure | 28.4% | *1.6%* | 31.0% | *2.0%* | 24.5% | *5.5%* | 22.8% | *2.8%* | 32.9% | *2.2%* | 25.4% | *2.7%* | 26.2% | *1.9%* | 30.1% | *1.8%* |
