# Supplementary material for: HIV-Related Discrimination among Grade Six Students in Nine Southern African Countries
Source: PLoS One. 2014 Aug 8;9(8):e102981. doi: 10.1371/journal.pone.0102981 (PMC4126685; doi:10.1371/journal.pone.0102981)
Supplement: Digital Content S3 — Table: Student responses to “Should a pupil who is infected with HIV be allowed to continue to attend school?” by sub-groups. (DOCX) [file pone.0102981.s003.docx]

Digital content S3, Table: Student responses to "Should a pupil who is infected with HIV be allowed to continue to attend school?" by sub-groups.

|  |  | **National** | *SE* | **Rural** | *SE* | **Small** **Town** | *SE* | **Large** **City** | *SE* | **Poorest 25%** | *SE* | **Richest 25%** | *SE* | **Boys** | *SE* | **Girls** | *SE* |
| --- | --- | --- | --- | --- | --- | --- | --- | --- | --- | --- | --- | --- | --- | --- | --- | --- | --- |
| **Botswana** | No | 26.4% | 1.3% | 31.8% | 1.9% | 20.9% | 3.1% | 21.6% | 1.6% | 37.8% | 2.2% | 15.8% | 2.1% | 28.0% | 1.6% | 24.7% | 1.5% |
|  | Yes | 50.1% | 1.6% | 44.2% | 2.2% | 58.7% | 4.3% | 53.2% | 2.5% | 37.9% | 2.3% | 63.2% | 2.2% | 48.9% | 1.9% | 51.2% | 1.8% |
|  | Not Sure | 23.6% | 1.1% | 24.0% | 1.6% | 20.4% | 2.9% | 25.1% | 1.8% | 24.3% | 1.6% | 21.0% | 1.6% | 23.1% | 1.4% | 24.0% | 1.3% |
| **Lesotho** | No | 37.2% | 2.0% | 41.4% | 2.8% | 34.3% | 3.2% | 23.2% | 3.8% | 42.1% | 2.5% | 28.8% | 2.6% | 39.0% | 2.4% | 35.8% | 2.0% |
|  | Yes | 43.8% | 2.0% | 41.6% | 2.7% | 42.2% | 3.1% | 54.5% | 4.5% | 40.8% | 2.8% | 51.8% | 2.7% | 42.9% | 2.1% | 44.6% | 2.1% |
|  | Not Sure | 18.9% | 1.2% | 17.0% | 1.4% | 23.4% | 3.2% | 22.3% | 3.1% | 17.0% | 1.7% | 19.4% | 2.2% | 18.1% | 1.4% | 19.7% | 1.4% |
| **Malawi** | No | 12.1% | 1.4% | 14.1% | 1.8% | 1.3% | 0.6% | 9.7% | 2.0% | 14.0% | 2.6% | 9.1% | 1.5% | 10.2% | 1.3% | 14.1% | 1.9% |
|  | Yes | 83.9% | 1.7% | 81.9% | 2.1% | 94.8% | 2.2% | 85.7% | 3.0% | 81.4% | 3.0% | 87.7% | 1.7% | 85.3% | 1.7% | 82.4% | 2.1% |
|  | Not Sure | 4.0% | 0.6% | 4.0% | 0.7% | 4.0% | 2.0% | 4.6% | 1.9% | 4.7% | 1.4% | 3.2% | 0.8% | 4.6% | 0.8% | 3.5% | 0.7% |
| **Mozambique** | No | 25.2% | 1.2% | 32.3% | 2.3% | 26.5% | 2.2% | 17.0% | 1.4% | 29.1% | 2.6% | 16.9% | 1.6% | 25.8% | 1.5% | 24.4% | 1.5% |
|  | Yes | 47.3% | 1.6% | 43.0% | 2.7% | 49.3% | 2.9% | 50.1% | 2.8% | 47.3% | 2.7% | 48.3% | 2.6% | 48.1% | 1.8% | 46.4% | 1.9% |
|  | Not Sure | 27.5% | 1.2% | 24.7% | 2.0% | 24.2% | 2.1% | 32.9% | 2.3% | 23.6% | 1.8% | 34.8% | 2.3% | 26.1% | 1.4% | 29.2% | 1.5% |
| **Namibia** | No | 25.8% | 1.3% | 29.2% | 1.7% | 22.3% | 2.1% | 18.7% | 2.7% | 28.8% | 2.2% | 17.4% | 1.7% | 26.3% | 1.4% | 25.3% | 1.4% |
|  | Yes | 50.5% | 1.5% | 48.4% | 1.9% | 50.2% | 2.7% | 57.6% | 3.5% | 50.3% | 2.4% | 59.6% | 2.3% | 51.1% | 1.5% | 49.9% | 1.7% |
|  | Not Sure | 23.7% | 1.0% | 22.5% | 1.4% | 27.5% | 2.0% | 23.7% | 2.0% | 20.9% | 1.6% | 22.9% | 1.5% | 22.5% | 1.1% | 24.8% | 1.2% |
| **South** **Africa** | No | 21.7% | 1.0% | 27.9% | 1.7% | 17.1% | 1.7% | 14.7% | 1.2% | 30.4% | 1.9% | 11.6% | 1.0% | 23.7% | 1.1% | 19.8% | 1.1% |
|  | Yes | 56.9% | 1.4% | 48.9% | 2.2% | 63.0% | 2.9% | 66.0% | 2.1% | 46.3% | 2.5% | 71.7% | 1.7% | 55.3% | 1.5% | 58.5% | 1.7% |
|  | Not Sure | 21.4% | 0.9% | 23.2% | 1.4% | 20.0% | 1.7% | 19.3% | 1.5% | 23.3% | 1.6% | 16.8% | 1.2% | 21.0% | 1.0% | 21.7% | 1.1% |
| **Swaziland** | No | 12.9% | 1.0% | 15.1% | 1.2% | 8.6% | 1.9% | 7.6% | 2.6% | 13.3% | 1.4% | 9.2% | 1.4% | 13.2% | 1.1% | 12.7% | 1.3% |
|  | Yes | 71.7% | 1.6% | 68.8% | 1.9% | 75.6% | 4.2% | 80.9% | 3.6% | 69.0% | 2.4% | 76.0% | 2.2% | 71.9% | 1.7% | 71.5% | 1.9% |
|  | Not Sure | 15.3% | 1.1% | 16.1% | 1.4% | 15.8% | 2.8% | 11.5% | 1.9% | 17.7% | 2.2% | 14.8% | 1.5% | 14.9% | 1.1% | 15.8% | 1.4% |
| **Zambia** | No | 33.4% | 1.8% | 35.1% | 2.3% | 34.1% | 4.3% | 27.2% | 4.0% | 36.8% | 2.9% | 25.3% | 2.2% | 31.9% | 2.0% | 34.9% | 2.1% |
|  | Yes | 55.6% | 2.0% | 54.9% | 2.4% | 54.1% | 4.8% | 58.9% | 5.1% | 51.2% | 3.2% | 62.1% | 2.8% | 57.8% | 2.2% | 53.3% | 2.3% |
|  | Not Sure | 11.0% | 0.9% | 10.0% | 1.0% | 11.8% | 2.3% | 13.9% | 2.2% | 12.1% | 1.6% | 12.6% | 1.5% | 10.3% | 1.1% | 11.8% | 1.1% |
| **Zimbabwe** | No | 41.7% | 2.1% | 49.7% | 2.4% | 28.7% | 6.5% | 16.9% | 2.3% | 51.5% | 2.7% | 20.1% | 2.4% | 41.7% | 2.7% | 41.7% | 2.2% |
|  | Yes | 43.6% | 2.2% | 36.6% | 2.4% | 52.4% | 8.8% | 66.3% | 3.4% | 35.5% | 3.0% | 63.6% | 3.3% | 44.8% | 2.8% | 42.7% | 2.2% |
|  | Not Sure | 14.7% | 1.1% | 13.6% | 1.3% | 18.9% | 3.5% | 16.9% | 2.0% | 12.9% | 1.8% | 16.3% | 2.0% | 13.5% | 1.4% | 15.6% | 1.3% |
